# Shared and specific patterns of structural and functional thalamo-frontal disturbances in manic and euthymic pediatric bipolar disorder

**DOI:** 10.1007/s11682-021-00539-z

**Published:** 2021-08-25

**Authors:** Yi-Bing Guo, Wei-Jia Gao, Zhi-Liang Long, Wei-Fang Cao, Dong Cui, Yong-Xin Guo, Qing Jiao, Jian-Feng Qiu, Lin-Yan Su, Guang-Ming Lu

**Affiliations:** 1 Faculty of Psychology, Southwest University, Chongqing, 400715 China; 2 Research School of Psychology, The Australian National University, Canberra, Australia; 3 Department of Child Psychology, The Children’s Hospital, Zhejiang University School of Medicine, Hangzhou, 310003 Zhejiang China; 4 Department of Radiology, Shandong First Medical University &Shandong Academy of Medical Sciences, Tai’an, 271016 Shandong Province China; 5 Institute of Biomedical Engineering, Chinese Academy of Medical Science & Peking Union Medical College, Tianjin, 300192 China; 6 Mental Health Institute of the Second Xiangya Hospital, Central South University, Changsha, 410011 China; 7 Department of Medical Imaging, Jinling Hospital, Clinical School of Medical College, Nanjing University, Nanjing, 210002 China

**Keywords:** Thalamo-frontal loop, Manic pediatric Bipolar disorder, Euthymic pediatric bipolar disorder, Functional connectivity, Functional magnetic resonance imaging

## Abstract

Bipolar disorder (BD) is clinically defined by alternating depressive and manic episodes with a separated period of euthymia. Thalamo-frontal loop plays vital role in psychotic symptoms, altered motor control and executive difficulties in BD. It remains unclear that structural and functional alterations of thalamo-frontal loop among the different mood states in BD, especially in pediatric BD(PBD).

Twenty manic PBD (mPBD), 20 euthymic PBD (ePBD) and 19 healthy controls (HCs) were included in the study. By analyzing the T1 images and fMRI signals, thalamus volume and frontal grey matter cortical thickness were tested, and functional connectivity (FC) between bilateral thalamus and frontal cortex was calculated. Relationship between clinical indices and thalamo-frontal FC was also evaluated in mPBD and ePBD adolescents.

Compared to HCs, the cortical thickness of left middle frontal gyrus (MFG), bilateral superior frontal gyrus (SFG) was significantly decreased in both mPBD and ePBD patients, and volume of left thalamus and cortical thickness of right MFG significantly decreased in mPBD patients. Compared to that of the HCs and ePBD subjects, thalamo-frontal hyperconnectivity with MFG was found in mPBD, and compared with that of HCs, thalamo-frontal hypoconnectivity with precentral gyrus/SFG was found in ePBD. In ePBD patients, episode times positively correlated with FC values between thalamus and precentral gyrus.

The findings of the present study demonstrate detailed knowledge regarding shared and specific structural and functional disruption in thalamo-frontal loop in mPBD and ePBD subjects. Thalamo-frontal abnormalities reported in adult BD subjects were also observed in adolescent BD patients, and thalamo-frontal dysfunction may be a crucial treatment target in BD.

## Introduction

Bipolar disorder is a psychiatric disorder characterized by severe intermittent mood disorders that vary between mania, depression, and euthymia(Altinay et al., 2016), affected about 3.9% of youth under age of 18(Van Meter, Moreira, & Youngstrom, 2019). Corticolimbic model, including ventrolateral circuit, ventromedial circuit, dorsolateral circuit and anterior cingulate circuit, had been put forward to explain internal and external emotional control along with cognition in bipolar disorder(Brooks & Vizueta, 2014). In this model, thalamus play a critical role through its bilateral projections with prefrontal cortex. Substantial evidence had elucidated that thalamus contributes to the pathogenesis of bipolar disorder(Duarte et al., 2016; Nery, Monkul, & Lafer, 2013). However, inconsistent results had been reported showing increased, decreased and no different volume and functional connectivity (FC) in thalamo-frontal loop in BD subjects.

Several studies suggested volumetric deficits of the thalamus in BD patients. For example, decreased grey matter volume in right thalamus and bilateral prefrontal cortex were reported in BD subjects(Hibar et al., 2018; Yu et al., 2019). Increased left thalamic volume in male manic BD was found compared to healthy controls(HCs)(Z. Chen et al., 2012). While patients with bipolar I disorder were found having no significant volumetric thalamus abnormalities during early stage of the disease(Arumugham et al., 2017). And no significant differences concerning the thalamus volume both in adult BD(L. Chen et al., 2018; Liberg et al., 2015) and pediatric BD(PBD) patients(Frazier et al., 2005) were also reported. On the context of frontal cortex, diminished grey matter of the prefrontal cortex in BD(Soloff et al., 2008) and larger grey matter volume of right inferior frontal gyrus in healthy offspring of BD parents(Hajek et al., 2013) had been reported. Disruptions of the structure may lead to dysfunction of the thalamo-frontal loop, which may present as common symptom of BD, such as failure in emotional regulation, altered motor control and executive difficulties. For instance, increased thalamic connectivity with orbitofrontal cortex has been demonstrated in BD adolescents(Ramsay, 2019), and in medication-free adolescents combining BD and attention deficit hyperactivity disorder(Son et al., 2017). In brief, accumulating evidence supported the structural and functional abnormalities of thalamo-frontal loop in BD patients. However, little attention has been paid to the structural and functional changes of thalamo-frontal loop among different mood states in BD, especially in pediatric BD. In general, compared with that of adult BD, PBD presents with more atypical symptoms and comorbidities, difficult to complete remission, more recurrences and longer disease durations(Goldstein & Birmaher, 2012). Investigation of thalamus and frontal cortex networks in manic PBD (mPBD) and eythymic PBD(ePBD) may be crucial for understanding the pathophysiology of PBD.

The aim of the current study is to explore the shared and specified structural and functional alterations of thalamo-frontal loop in mPBD and ePBD. Based on previous findings, we predicted that both euthymia and manic patients with PBD would present abnormalities in thalamic and frontal structure and thalamo-frontal FC. While testing this hypothesis, the issue of thalamus volume and frontal grey matter cortical thickness were tested and FC between bilateral thalamus and frontal cortex was calculated. Relationship between clinical indices and thalamo-frontal FC was also evaluated in mPBD and ePBD subjects.

## Methods

### Subjects

The data of the present study was extracted from January 2012 to July 2014. Forty PBD patients (20 manic PBD and 20 euthymic PBD) were included in the present study, who were recruited from the Child and Adolescent Psychiatry Clinic of the Second Xiangya Hospital of Central South University in Changsha, Hunan. Nineteen age- and gender-matched healthy controls were recruited from local middle schools.

The criteria for the inclusion of PBD patients were as follows: 1) meeting Diagnostic and Statistical Manual for Mental Disorders, Fourth Edition (DSM-IV) criteria for bipolar disorder (BD), including hypomania (≥ 4 days) or mania (≥ 7 days) that met at least one full DSM-IV-TR criteria; 2) may be instructed to hold body still during MRI scanning; 3) Han nationality; 4) 10–18 years old; 5) right handedness. Exclusion criteria for all participants included: 1) IQ < 80; 2) with alcohol or drug abusing or dependence; 3) with other mental disorders, such as schizophrenia or autism; 4) with major sensor motor handicaps. 5) with contraindications to MRI scans, including claustrophobia, stents, and metal implants.

All procedures in the present study were in accordance with the ethical standards of Xiangya Hospital and the National Research Council. Written informed consents were obtained from all the subjects and their guardian.

### Clinical evaluations

By employing Schedule for Affective Disorders and Schizophrenia for School aged Children Present and Lifetime Versions (K-SADS-PL)(Kaufman et al, 1997), two experienced pediatric psychiatrists determined the diagnosis of all PBD patients. Criteria for diagnosis of manic PBD patients were DSM-IV criteria for mania or hypomania but were not met DSM-IV criteria for depressive symptom. Meanwhile, euthymic PBD subjects were not met DSM-IV criteria for mania, depression, hypomania or dysthymia, and should be in remission state for at least four weeks. All participants' intelligence was assessed by the Wechsler Abbreviated Scale of Intelligence (WASI). The severity of emotional symptoms of all of the subjects were assessed using Mood and Feelings Questionnaire (MFQ)(Chang et al., 2005) and Young Mania Rating Scale (YMRS)(Young et al., 1978) prior to MRI scanning. MFQ has 33 items, which is a well-designed measure to evaluate emotional symptoms of subjects. People with more severe of depression have a higher score of MFQ. YMRS has 11 items and is a clinician rating scale. YMRS scores were associated with manic symptoms, with higher scores indicating more manic symptoms. Some clinical information such as onset age, illness duration, episode times, psychotic symptoms and so on were recorded.

### MRI data acquisition and preprocessing

MRI images were acquired on a 3.0 T Siemens Trio scanner (Siemens, Munich, Germany). During the MRI scanning, foam pads were placed on both sides of subjects' heads to limit head movement. And cotton plugs were used to reduce noise and protect subjects’ hearing. Each subject was told to stay awake, keep their eyes closed and not think about anything in particular during the MRI scanning. The subjects were asked after the MRI scanning if they had fallen asleep in the scanner. If a subject did not keep awake during the scan, the data was excluded. T1-weighted images were acquired by employing three-dimensional magnetization-prepared rapid acquisition gradient echo (3D MPRAGE) protocol. Detailed sampling parameters covering the entire brain include repetition time (TR) = 2300 ms, echo time (TE) = 2.03 ms, flip angle = 9°, inversion time = 900 ms, field of view (FOV) = 256 mm*256 mm, matrix = 256*256, thickness = 1 mm and gap = 0 mm. Resting-state fMRI data were collected by plane echo imaging sequence. The detailed parameters were set as: 30 axial slices, TR = 2000 ms, TE = 30 ms, FOV = 240 mm*240 mm, gap = 0.4 mm, slice thickness = 4 mm, flip angle = 90 ^o^, matrix = 64*64 and scanning time is 500 s.

FreeSurfer 6.0 software (http://surfer.nmr.mgh.harvard.edu/) was used for T1-weighted images processing. Brain volumetric calculation were conducted by recon stream (“recon-all”). The procedure including correction, skull stripped, intensity normalization, automated Talairach transformation, gray/white matter tessellation, and topology correction(Fischl et al., 2001). The segmented subcortical structures were examined by a nonlinear warping atlas, and then the subcortical structure volumes were obtained, including thalamus, caudate, putamen, amygdala, hippocampus, pallidum, and accumbens. Grey matter cortical thickness values were calculated with 68 (34 regions for each hemisphere) cortical regions based on Desikan-Killiany atlas(Desikan et al., 2006). The bilateral thalamus volumes and frontal grey mater cortical thickness of each subject were extracted for further research.

MRI images were preprocessed by Data Processing and Analysis of Brain Imaging (DPABI V5.1, http://rfmri.org/dpabi Yan et al., 2016). Resting-state fMRI signals were processed in the following steps. To remove the T1 saturation effect, the first 10 volumes of fMRI images were discarded. The images were then sectioned for slice timing, realignment, nuisance covariates regression (Friston 24-parameter model, white matter signals, cerebrospinal fluid signals and global signals) and spatial normalization (3*3*3 mm^3^). Subjects with exceeded head motion (more than 2.0 mm translation or 2.0° rotational movements) will be excluded from further research. Data were band-pass filtered, preserving frequencies between 0.01 and 0.08 Hz. The fMRI images were further spatial smoothed using a 6 mm Gaussian kernel of FWHM. Finally, scrubbing was performed for motion correction to reduce the negative influence in functional connectivity(Power et al., 2012).

### Functional connectivity analysis

Bilateral thalamus from Automated Anatomical Labeling (AAL) parcellation template(Tzourio-Mazoyer et al., 2002) were defined as seeds of regions of interest (ROIs). For each subject, the time series of the bilateral thalamus were extracted, and Pearson correlation coefficients between fMRI signals of the thalamus and that of all voxels of the brain were calculated. These r values were then converted to z values using Fisher r-to-z transformation to generate a map of functional connectivity. For the purpose of investigating relation between thalamus and frontal cortex, FC maps of the whole brain was masked into frontal cortex with a frontal mask from AAL template. Signals of the brain regions showing significant difference in FC among the three groups were extracted and further analyzed for post hoc T test between groups.

## Statistical analysis

Statistical analysis was performed using IBM SPSS software (version 25.0, Armonk, NY, United States). The classification variables (gender, psychotic symptoms, types of bipolar disorder and family history) were tested by Pearson chi-square test. One-way analysis of variance (ANOVA) was used for testing group differences of continuous variables, thalamic volumes and frontal grey matter thickness among the three groups (total intracranial volume (TIV) regressed) and two-sample T test was used for post hoc between-group multiple comparison (TIV regressed). Correlations between clinical scores and thalamic volumes/frontal grey matter thickness in the patient groups were calculated with total intracranial volume regressed. Due to the small sample of each group in the current study, effect size (Cohen’s d) was calculated to disclose between-group difference of volume/thickness and FC.

Difference of thalamo-frontal FC among mPBD, ePBD patients and HCs was analyzed by ANOVA. Gaussian random field’s (GRF) theory was performed for correcting process, with voxel-wised p < 0.005 and cluster-wised p < 0.05. The brain regions showing significant differences among the three groups were identified as ROIs. The average FC value of each ROIs was extracted for subsequent two-sample T test analysis to compare the between-group FC values difference. Bonferroni correction was used for controlling false positive rate. In PBD group, voxel-wised correlation analysis was conducted between clinical indices and thalamo-frontal FC values with age and gender regressed (GRF corrected, with voxel-wised p < 0.005 and cluster-wised p < 0.05).

## Results

### Demographic and clinical data

No subject had fallen asleep during the MRI scanning. As displayed in Table 1, there was no significant differences in age(p = 0.080), gender(p = 0.684), years of education(p = 0.259), IQ(p = 0.085) and MFQ scores(p = 0.738) among mPBD group, ePBD group and HCs. The difference of YMRS score(p < 0.001) among the three groups was statistically significant. Meanwhile, no significant difference was found between mPBD and ePBD patients in onset age(p = 0.410), illness duration(p = 0.065), episode times(p = 0.360), psychotic symptoms(p = 0.342), type of bipolar disorder(p = 0.168), and familial BD history(p = 0.736).

**Table 1:** Demographic characteristics and clinical variables

Characteristics	mPBD(n = 20)	ePBD(n = 20)	HCs(n = 19)	Statistic(F/T/χ2)	p-value
Gender (male/female)	8/12	10/10	7/12	0.76^c^	0.684
Age (years)	15.10 ± 1.65	15.30 ± 1.72	14.16 ± 1.57	2.641^a^	0.080
Education (years)	8.35 ± 1.76	8.40 ± 1.85	7.47 ± 2.22	1.385 ^a^	0.259
IQ	100.10 ± 13.56	107.50 ± 9.62	105.32 ± 7.51	2.579 ^a^	0.085
YMRS scores	34.60 ± 6.32	5.35 ± 1.60	3.63 ± 2.06	380.3 ^a^	< *0.001*
MFQ scores	7.20 ± 2.59	6.60 ± 4.44	6.37 ± 3.00	0.305 ^a^	0.738
Onset age (years)	14.00 ± 1.69	13.50 ± 2.09		0.833^b^	0.410
Illness duration (months)	13.65 ± 11.46	22.55 ± 17.56		-1.898 ^b^	0.065
Episode times	3.05 ± 1.70	4.65 ± 7.53		-0.926 ^b^	0.360
Psychotic symptoms (yes/no)	9/11	12/8		0.902 ^c^	0.342
BD-I/BD-II	16/4	12/8		1.905 ^c^	0.168
Familial BD history (yes/no)	7/13	6/14		0.114 ^c^	0.736
Medications					
Lithium(n/%)	9/45%	7/35%			
Valproate(n/%)	11/55%	15/75%			
Atypical antipsychotics(n/%)	14/70%	17/85%			
Antidepressants(n/%)	1/5%				

^a^ one-way ANOVA; ^b^ Two-sample t-test; ^c^ chi-square test.

Values are presented as mean ± standard deviation.

mPBD: manic pediatric bipolar disorder; ePBD: euthymic pediatric bipolar disorder; HCs: healthy controls; IQ: intelligence quotient; YMRS: Young Manic Rating Scale; MFQ: Mood and Feelings Questionnaire; BD-I, bipolar disorder type I; BD-II, bipolar disorder type II.

### Difference of thalamic volume and frontal cortical thickness among groups

As shown in Table 2, volume of the left thalamus (THA), grey matter cortical thickness of bilateral opercular inferior frontal gyrus (IFG.ope), middle frontal gyrus (MFG) and superior frontal gyrus(SFG) showed differences among the three groups. Post hoc analysis exhibited that compared with that of the HCs, the volume of left thalamus and cortical thickness of right MFG significantly decreased in mPBD patients, cortical thickness of left MFG, left SFG and right SFG was significantly decreased in both mPBD and ePBD patients (p < 0.05, Bonferroni corrected).

**Table 2:** Regions showing differences in thalamic volume and frontal grey matter thickness among mPBD, ePBD patients and HCs

Region	Main effect				Post Hoc						
			mPBD vs. ePBD		mPBD vs. HCs			ePBD vs. HCs			
	F	p	T	p	Cohen’d	T	p	Cohen’d	T	p	Cohen’d
THA.L	6.184	0.004**	-1.790	0.237	-0.589	-3.516	0.003**	-1.044	-1.749	0.257	-0.585
THA.R	2.474	0.093									
IFG.ope.L	3.934	0.025*	-0.062	1.000	-0.02						
IFG.orb.L	2.569	0.086									
IFG.tri.L	3.091	0.053									
MFG.L	4.906	0.011*	-0.37	1.000	-0.12	-2.892	0.016*	-0.95	-2.517	0.044*	-0.83
SFG.L	6.585	0.003**	-0.005	1.000	-0.001	-3.159	0.008**	-1.04	-3.153	0.008**	-1.04
PreCG.L	2.441	0.096									
IFG.ope.R	3.691	0.031*	-0.939	1.000	-0.30	-2.681	0.029*	-0.88	-1.754	0.255	-0.57
IFG.orb.R	0.405	0.669									
IFG.tri.R	2.083	0.134									
MFG.R	3.555	0.035*	-1.280	0.617	-0.42	-2.666	0.030*	-0.87	-1.401	0.499	-0.46
SFG.R	4.264	0.019*	-0.128	1.000	-0.04	-2.601	0.036*	-0.85	-2.473	0.049*	-0.81
PreCG.L	2.131	0.128									

mPBD: manic pediatric bipolar disorder; ePBD: euthymic pediatric bipolar disorder; HCs: healthy controls; THA.L: left thalamus; THA.R: right thalamus.

Presented adjusted p < 0.05 was considered to indicate a significant difference (Bonferroni corrected).

**p < 0.01, *p < 0.05.

IFG.ope: opercular inferior frontal gyrus; IFG.orb: orbital inferior frontal gyrus; IFG.tri: triangular inferior frontal gyrus; MFG: middle frontal gyrus; SFG: superior frontal gyrus.

### Differences in thalamo-frontal FC

**Fig. 1:**
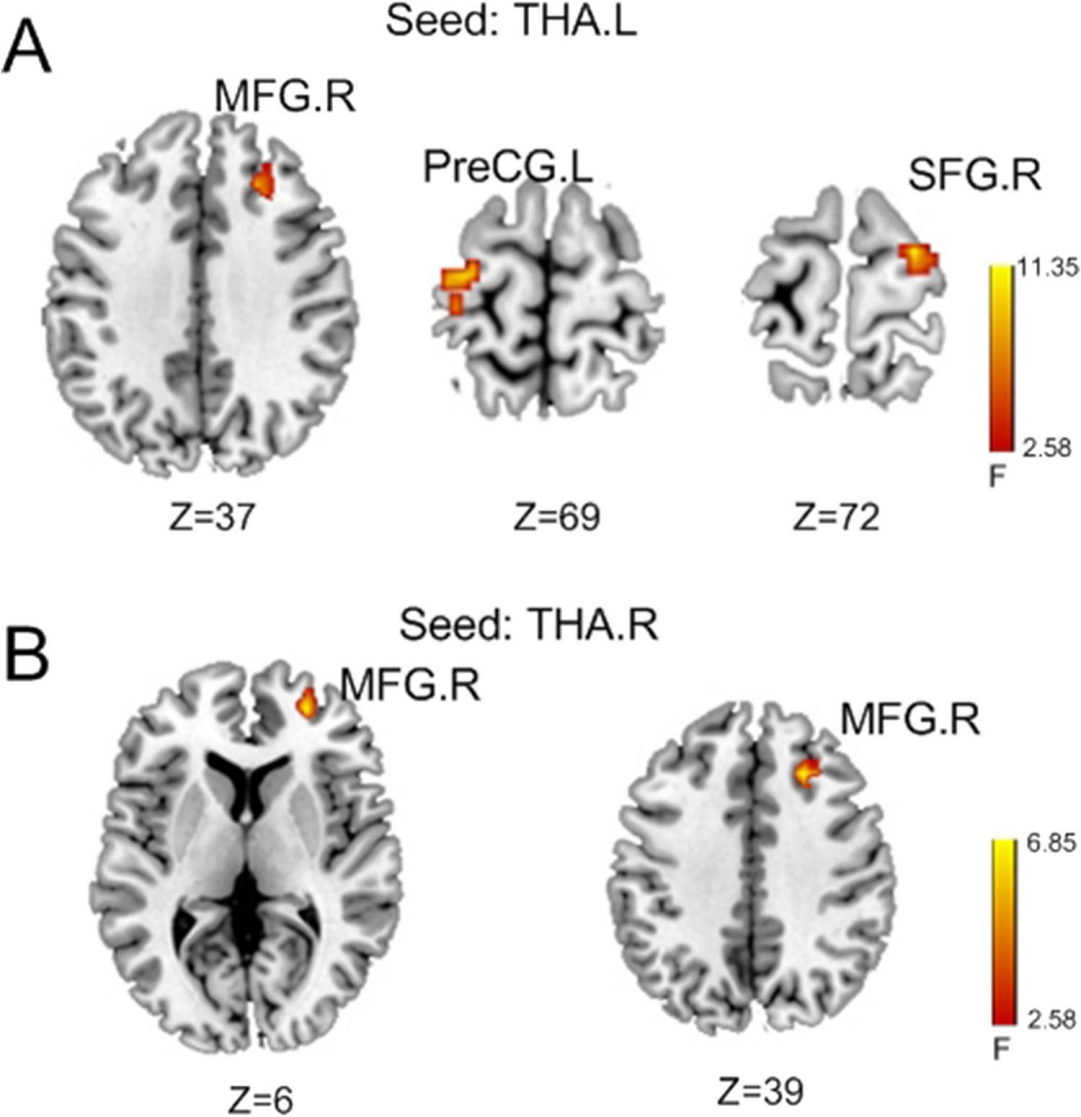
Statistical difference maps showing difference in thalamo-frontal FC among mPBD, ePBD patients and HCs by ANOVA analysis. (p < 0.05, GRF corrected). THA.L: left thalamus; THA.R: right thalamus; MFG: middle frontal gyrus; SFG: superior frontal gyrus; PreCG: precentral gyrus

**Fig. 2:**
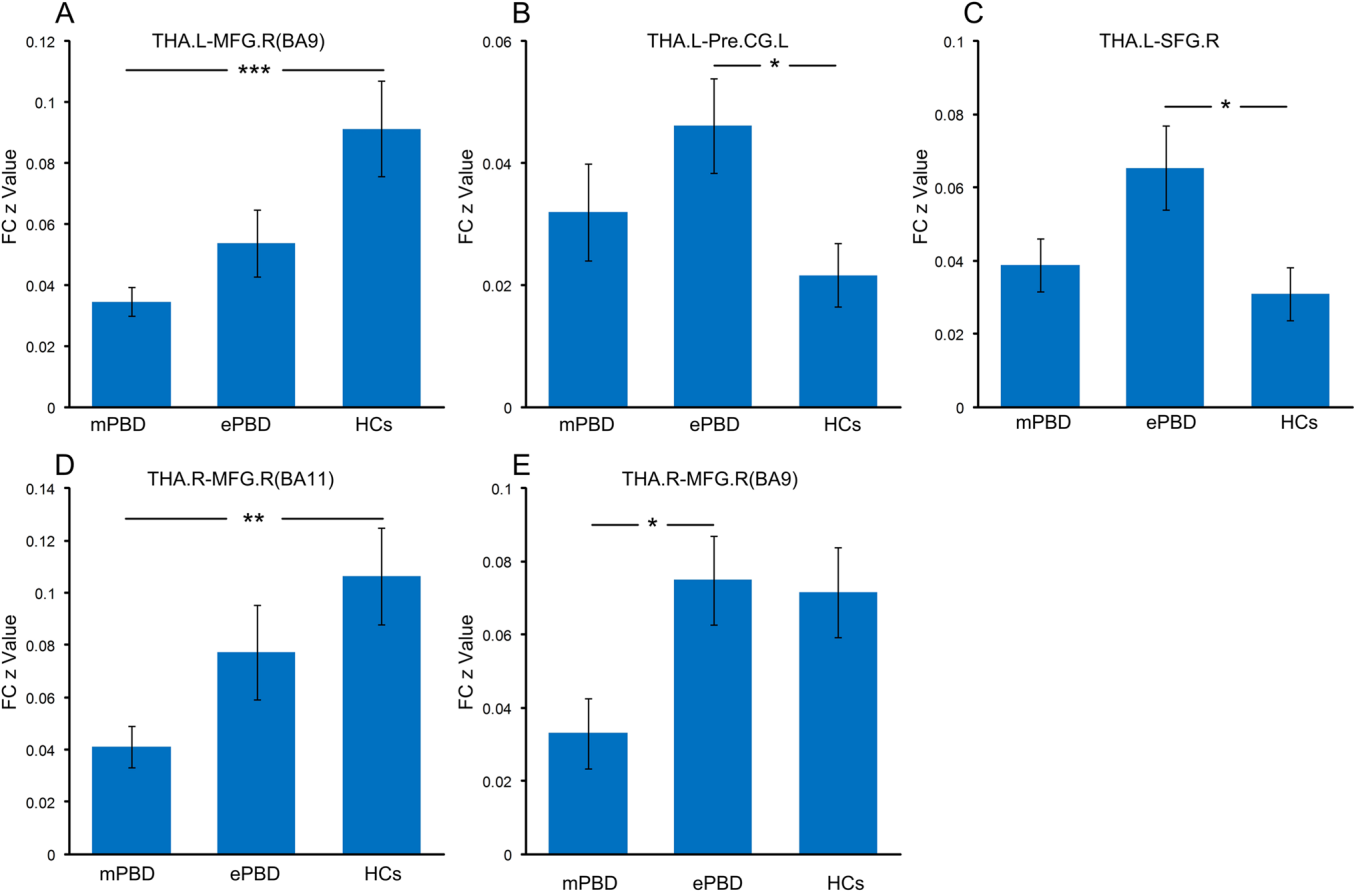
Pair wise comparison of z values of thalamo-frontal FC in mPBD, ePBD and HCs groups. (Two-sample T test; p < 0.05, Bonferroni corrected) mPBD: manic pediatric bipolar disorder; ePBD: euthymic pediatric bipolar disorder; HCs: healthy controls; L: left; R: right; SFG: superior frontal gyrus; MFG: middle frontal gyrus; PreCG: precentral gyrus; BA: Brodmann area *: p < 0.05; **: p < 0.01; ***: p < 0.001

As a result of ANOVA on resting-state FC of mPBD, ePBD and HCs groups, there were significant differences in FC between left thalamus and right MFG, left precentral gyrus (PreCG) and right SFG, and FC between the right thalamus and right MFG(Fig. 1). These brain regions were detailed in Table 3. The fMRI signals of the above areas were extracted for further post hoc tests. As shown in Fig. 2, in mPBD group, FC between left THA and right MFG (BA9, BA11) was reduced compared with that of the HCs (Fig. 2A,2D), and FC between left THA and right MFG(BA9) was reduced compared with that of the ePBD subjects (Fig. 2E). In ePBD group, FC between left THA and left PreCG, and right SFG were significantly reduced compared with that of the HCs (Fig. 2B,2C) (p < 0.05, Bonferroni corrected).

**Table 3:** Regions showing difference in thalamo-frontal FC among mPBD, ePBD patients and HCs

	Brodmann Area	MNI Coordinates X Y Z	Peak F value	Cluster size
*Seed:THA.L*				
MFG.R SFG.R PreCG.L	9 6 6	25 30 37 24 -9 72 -30 -15 72	8.63 8.17 5.32	71 62 63
*Seed:THA.R*				
MFG.R	11	27 51 6	6.78	57
MFG.R	9	24 27 39	6.27	58

mPBD: manic pediatric bipolar disorder; ePBD: euthymic pediatric bipolar disorder; HCs: healthy controls; L: left; R:right; MNI, Montreal Neurological Institute; SFG: superior frontal gyrus; MFG: middle frontal gyrus; PreCG: precentral gyrus.

### Correlation analysis between clinical indices and FC in PBD

**Fig. 3:**
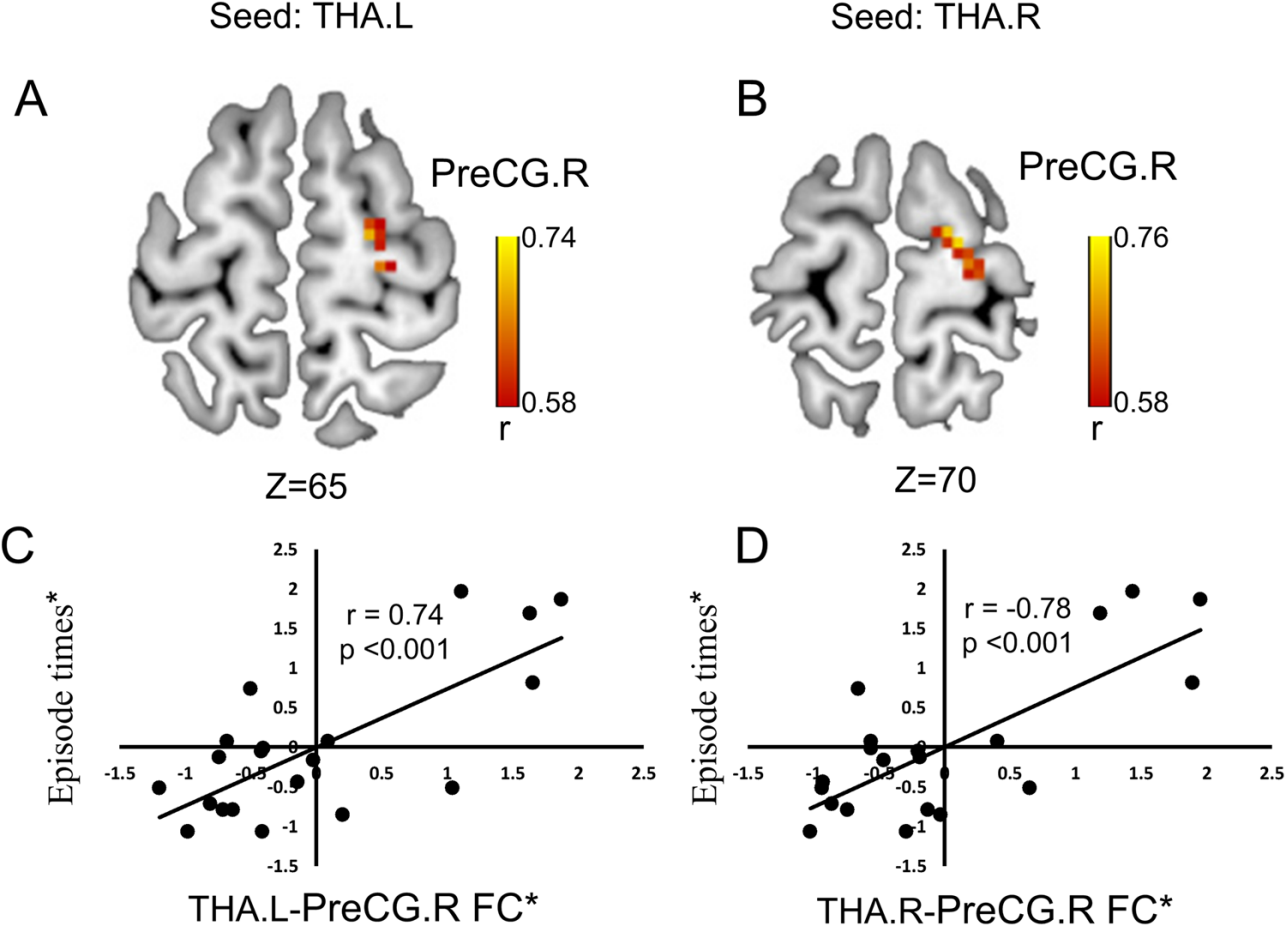
Voxel-wised correlation analysis between episode times and thalamo-frontal FC values in euthymic PBD. Episode times positively correlated with FC between THA.L and PreCG.R(A, C) and FC between THA.R and PreCG.R(B, D) (GRF corrected, voxel p < 0.005, cluster p < 0.05). Asterisk (*) represents the adjusted values with age and gender regressed. PreCG: precentral gyrus; L: left; R: right

In the present study, no significant correlation was found between the clinical indices and thalamic volume and frontal grey matter thickness in bipolar disorder patients. Voxel wised correlation analysis was conducted between clinical indices and thalamo-frontal FC values, and the significant results were exhibited in Fig. 3. In ePBD patients, episode times positively correlated with FC between left THA and right PreCG(BA6) (Fig. 3A,3C) and FC between right THA and right PreCG(BA6) (Fig. 3B,3D). (GRF corrected, voxel p < 0.005, cluster p < 0.05).

## Discussion

The goal of the present study is to explore alterations in volume of the thalamus, cortical thickness of the frontal regions and thalamo-frontal FC by analyzing T1 and BOLD signals of manic PBD, euthymic PBD and HCs. Several observations were obtained. First, both mPBD and ePBD subjects exhibited reduction in grey matter cortical thickness in the two groups include left MFG and bilateral SFG. While decreased volume of left thalamus and decreased cortical thickness of right IFG.ope and right MFG were only found in mPBD. Second, FC between the bilateral thalamus and MFG significantly decreased in mPBD patients compared with that of the HCs and ePBD subjects. FC between thalamus and SFG/PreCG was increased in ePBD compared to HCs. Third, in ePBD group, episode times was significantly positively correlated with the FC between thalamus and SFG/PreCG. These findings provide new evidence for shared and specific structural and functional connectivity disruptions, which may help to disclose neuropathological mechanisms underlying subtypes of PBD.

We found volume reduction in the left thalamus and gray matter cortical thickness reduction of frontal cortex in PBD subjects. It had been indicated that frontal-subcortical areas was early involved in BD development(Sigitova et al., 2017). The thalamus serves as a relay station within forebrain circuits to the cerebral cortex and to limbic structures(Jones, 1997). Significantly lower bilateral thalamic volumes in BD (Hibar et al., 2016) was demonstrated. No thalamic volume alteration were detected in ePBD subjects in the present study, which consisting with prior results(Radenbach et al., 2010).The prefrontal cortex plays a key role in “top-down” inhibitory control of internal and external sensory driven compulsive behaviors. Some studies had reported grey matter reduction of the prefrontal cortex(Soloff et al., 2008; Yu et al., 2019), which may contribute to the dysregulation of affect and emotion in BD(Soloff et al., 2008). The grey matter of the human cerebral cortex is made up of neurons and glial cells. Neurotrophins, which was found to be related with formation of new neurons or glial cells in animal models, was confirmed to decrease in manic and depressive of BD(Duman & Monteggia, 2006). Meanwhile, significantly reduced concentrations of phosphocreatine in grey matter in manic and euthymic bipolar I disorder subjects had also been reported. Phosphocreatine is accounted for decreased neuronal cell density or impaired oxidative phosphorylation(Dudley et al., 2016). Taken together, these factors may play an important role in brain cell development and may induce abnormal development of a brain area, which may cause the grey matter volume reduction of the thalamus and frontal cortex in PBD. Reduction of cortical thickness of the same prefrontal regions in the mPBD and ePBD subjects may suggest that dysfunction of the prefrontal cortex persist across different mood states.

In the present study, there is no shared alteration of thalamo-frontal connections in the mPBD and ePBD subjects. In manic PBD, aberrant thalamo-prefrontal connections was characterized by decreased thalamo-prefrontal FC(PFC) with right MFG (BA9, BA11) compared to HCs, and with right MFG(BA9) compared to ePBD. The results are consistent with previous study showing thalamo-PFC under-connectivity in bipolar disorder subjects(Anticevic et al., 2014). In a study combining schizophrenia and bipolar I disorder, reduced PFC-thalamic connectivity was confirmed in both chronic and early-stage psychosis patients(Woodward & Heckers, 2016). In addition, circuits in corticolimbic model(Brooks & Vizueta, 2014), was contributed to some clinical features of bipolar disorder(Strakowski et al., 2012). PFC areas in these circuits, including dorsolateral PFC(DLPFC)(BA9) and ventromedial PFC(VMPFC)(BA11), both have reciprocal projections with the thalamus. The DLPFC, playing an essential role in mood regulation, decision making and working memory, exhibited decreased metabolism in mania(Gonul et al., 2009). The MPFC is related to self-referential thought and reviewing past knowledge for preparing future action. In a mouse experiment, interaction between mediodorsal thalamus and MPFC was verified to play a key role in supporting working memory maintenance(Bolkan et al., 2017). The current findings of reduction of thalamic FC with DLPFC and VMPFC in mPBD may indicate a deficit in bottom-up attention and emotion processing, and the thalamofrontal feedback loops may be an important pathophysiological marker in PBD.

In euthymic PBD of the present study, thalamo-frontal connections with left PreCG (BA6) and right SFG(BA6) were increased compared with that of HCs. Meanwhile, episode times of historical manic or depressive symptom positively correlated with the FC values between bilateral thalamus and right left PreCG (BA6). The precentral gyrus is on the lateral surface of each frontal lobe, and it is the location of primary motor cortex which accounts for voluntary motor movement controlling. As a complicated sensory information node, the thalamus receives and processes sensory and motor input signals. The thalamus and precentral gyrus were reported to exhibit abnormality in ePBD patients. During emotion labeling condition, right thalamus showed decreased activation in remitted BD patients(Foland-Ross et al., 2012). Relative to healthy controls, remitted BD subjects demonstrated increased activation of PreCG in two-back task(Monks et al., 2004). Meanwhile, thalamocortical hyperconnectivity in sensory regions including motor visual, and occipital cortex has been characterized in BD (Ramsay, 2019). Hyperconnectivity between thalamus and precentral gyrus(BA6) was found in early stage of psychosis including schizophrenia and BD, which was interpreted as refinement of somatomotor-thalamic connectivity(Woodward & Heckers, 2016). Remitted BD subjects was verified showing increased thalamic connectivity with bilateral sensory-motor cortices, which may reflecting sensory gating disruption(Anticevic et al., 2014). Thalamic over-connectivity with primary motor cortex in ePBD in the present study is consistent with the above prior studies. Thalamo-primary motor cortex disturbances was only found in euthymic PBD, not in manic PBD subjects, which seems that the observed disturbances might somewhat be stable and be not altered or worsen during acute psychosis. However, the positively correlation between the thalamic-primary motor cortex connectivity and historical manic or depressive episode times may suggest the altered connectivity between thalamus and primary motor cortex also associated with historical symptom exacerbation of PBD.

## Limitations

The present study also has several limitations. Firstly, the small sample size may not large enough to detect volumetric or FC differences among groups completely, more data will be collected in future work to make the research more convinced. Secondly, all PBD patients in the current study received medication therapy, which may influence on interpreting the present result. Although researches have shown no effect of medication in neural differences between patient and controls(Jiang et al., 2020), future studies need to verify findings of the current study in patients without medication. Thirdly, the FC was investigated between the whole thalamus and all of the voxels of the frontal cortex. The approach provides better specificity within the frontal cortex but may obscure probing specific abnormalities of the nuclei of the thalamus. Actually studies had indicated a fatal role of mediodorsal thalamus in sustaining prefrontal activity during working memory maintenance(Bolkan et al., 2017) and altered mediodorsal thalamus activity may disrupt prefrontal-dependent cognitive behaviors(Parnaudeau et al., 2013). Future work would investigate structural and functional alterations of sub-nuclei of thalamus in PBD rather treating the thalamus as a homogeneous structure.

## Conclusions

The findings of the present study demonstrated detailed knowledge regarding shared and specific structural and functional alterations in thalamo-frontal loop in manic PBD and euthymic PBD subjects. The thalamo-frontal abnormalities reported in adult BD subjects are also observed in adolescent patients, and thalamo-frontal dysfunction may be a crucial treatment target in BD.
